# Glypican 6 Enhances *N*-Methyl-D-Aspartate Receptor Function in Human-Induced Pluripotent Stem Cell-Derived Neurons

**DOI:** 10.3389/fncel.2016.00259

**Published:** 2016-11-15

**Authors:** Kaoru Sato, Kanako Takahashi, Yukari Shigemoto-Mogami, Kaori Chujo, Yuko Sekino

**Affiliations:** Division of Pharmacology, Laboratory of Neuropharmacology, National Institute of Health SciencesTokyo, Japan

**Keywords:** human induced pluripotent stem cell, neuron, glypican 6, L-glutamate, *N*-methyl-D-aspartate receptor, membrane localization, excitotoxicity

## Abstract

The *in vitro* use of neurons that are differentiated from human induced pluripotent stem cells (hiPSC-neurons) is expected to improve the prediction accuracy of preclinical tests for both screening and safety assessments in drug development. To achieve this goal, hiPSC neurons are required to differentiate into functional neurons that form excitatory networks and stably express *N*-methyl-D-aspartate receptors (NMDARs). Recent studies have identified some astrocyte-derived factors that are important for the functional maturation of neurons. We therefore examined the effects of the astrocyte-derived factor glypican 6 (GPC6) on hiPSC-neurons. When we pharmacologically examined which receptor subtypes mediate L-glutamate (L-Glu)-induced changes in the intracellular Ca^2+^ concentrations in hiPSC neurons using fura-2 Ca^2+^ imaging, NMDAR-mediated responses were not detected through 7 days *in vitro* (DIV). These cells were also not vulnerable to excitotoxicity at 7 DIV. However, a 5-days treatment with GPC6 from 3 DIV induced an NMDAR-mediated Ca^2+^ increase in hiPSC-neurons and increased the level of NMDARs on the cell surface. We also found that GPC6-treated hiPSC-neurons became responsive to excitotoxicity. These results suggest that GPC6 increases the level of functional NMDARs in hiPSC-neurons. Glial factors may play a key role in accelerating the functional maturation of hiPSC neurons for drug-development applications.

## Introduction

Cellular reprogramming, in which terminally differentiated somatic cells are converted into pluripotent stem cells (induced pluripotent stem cells, i.e., hiPSCs) (Takahashi et al., 2007) is not only clinically relevant but also useful in drug development because these cells allow for the homogeneous derivation of mature human cell types in large quantities without the ethical issues associated with embryonic stem cells. In particular, the *in vitro* use of neurons differentiated from hiPSCs (hiPSC-neurons) at the preclinical drug development stage, including assessing pharmacological safety and searching therapeutic agents, is expected to improve the prediction accuracy of the effects of new candidate compounds in humans. To this end, it is required that hiPSC-neurons differentiate to functional neurons that form excitatory networks and stably express N-methyl-D-aspartate receptors (NMDARs). NMDARs have been implicated as mediators of excitotoxicity (Ferreira et al., 1996; Stout et al., 1998) and increased attention has been placed on the possibility of being the therapeutic target of psychiatric disorders such as schizophrenia (Field et al., 2011). Huge numbers of astrocytes are generated just prior to the postnatal wave of synaptogenesis, and astrocyte-derived factors play important roles in the functional differentiation of neurons in the developing brain (Wang and Bordey, 2008). Indeed, recent reports have identified some astrocyte-derived factors that are important for synaptic maturation (Christopherson et al., 2005; Allen et al., 2012). Allen et al. (2012) demonstrated that glypicans (GPCs) 4 and 6 induce functional synaptogenesis in retinal ganglion cells (RGCs). GPCs 4 and 6 are members of the 6 glycosylphosphatidylinositol (GPI)-anchored heparan sulfate proteoglycan (HSPG) family, and GPC6 is most homologous to GPC4 (Veugelers et al., 1999). In the present study, we examined the effects of GPC6, which is a commercially available GPC, on hiPSC-neurons. We found that a 5-days treatment with GPC6 from 3 days *in vitro* (DIV) increased the level of functional NMDAR expression on the cell surface. We also found that GPC6-treated hiPSC-neurons became vulnerable to excitotoxicity, which is a critical contributor to neuronal injury in several acute and chronic neurodegenerative disorders (Kritis et al., 2015). Glial factors may therefore play a key role in accelerating the functional maturation of hiPSC neurons for drug-development applications.

## Materials and Methods

### hiPSC-Derived Neuron Culture

Commercially available hiPSC-neurons were used in this study [iCell Neuron: Cellular Dynamics International (CDI), Madison, WI, USA] and were cultured according to the manufacturer’s instructions. The cells were plated in 8-well glass chambers (155409JP, Nunc, Waltham, MA, USA), 3.5-cm culture dishes, or 96-well plastic plates, which were pre-coated with poly-D-lysine (50 μg/ml for 24 h, P6407, Sigma, Darmstadt, Land Hessen, Germany) followed by a top coating with laminin (5 μg/ml for 2 h, L2020, Sigma, Darmstadt, Land Hessen, Germany), at a density of 75,000 cells/cm^2^ and cultured in iCell Neuron-maintenance medium (CDI). The medium was changed 24 h after plating and after every 3 days. The reproducibility of all experimental data was confirmed by three independent experiments using iCell Neurons with different lot numbers (#139991, #1361234, #1360614). The all experiments using hiPSC-neurons were approved by the Research Ethics Committee of National Institute of Health Sciences (NIHS) in accordance with the Declaration of Helsinki.

### Immunocytochemistry

After fixation with 4% paraformaldehyde (PFA) at room temperature for 30 min, hiPSC-neurons were permeabilized with 0.1% (vol/vol) Triton X-100 for 5 min, followed by treatment with blocking solution (5% [vol/vol] goat serum and 1% [wt/vol] BSA in PBS) for 60 min. After blocking, the cells were incubated with primary antibody (mouse monoclonal anti-β3 tubulin antibody [1:500] [MMS-435P, Covance, Princeton, NJ, USA], chicken polyclonal anti-GFAP antibody [1:400] [ab4674, Abcam, Cambridge, UK], rabbit polyclonal anti-Nestin antibody [1:200, AB5922, Millipore, Land Hessen, Germany], or chicken polyclonal anti-MAP2 antibody [1:5000, AB5392, Abcam, Cambridge, UK]) in blocking solution at 4°C for 24 h. After washing, the cells were incubated with solution containing Alexa Fluor 488-conjugated secondary antibody (1:500) (Invitrogen, Waltham, MA, USA) and Hoechst 33342 (1:200) (34607951, Dojindo, Kumamoto, Japan) at room temperature for 3 h. Cultured rat hippocampal neurons were counted in fluorescence images obtained and analyzed on a Nikon A1 confocal microscope system (Nikon, Tokyo, Japan).

### GPC6 Treatment

A stock solution of GPC6 (R&D systems, Minneapolis, MN, USA) (1.55 μM in PBS) was diluted to 10 nM with culture medium at the time of use. hiPSC-neurons were treated with GPC6 for 5 days from 3 DIV.

### Ca^2+^ Imaging

hiPSC-neurons or rat hippocampal neurons cultured on eight well-glass chambers were used for Ca^2+^ imaging. Changes in intracellular Ca^2+^ concentrations ([Ca^2+^]i) in single cells were measured using fura-2 as described previously (Koizumi and Inoue, 1997), with minor modifications. Briefly, the culture medium was replaced with balanced salt solution (BSS) with the following composition (mM): NaCl 150, KCl 5.0, CaCl_2_ 1.8, MgCl_2_ 1.2, *N*-2-hydroxyethylpiperazine-*N*′-2-ethanesulfonic acid (HEPES) 25, and D-glucose 10 (pH = 7.4). The cells were loaded with 10 μM fura-2 acetoxymethylester (fura-2 AM) (Dojindo Molecular Technology, Inc., Kumamoto, Japan) in BSS at room temperature for 45 min and washed with BSS. The glass chamber was mounted on an inverted epifluorescence microscope (TE-2000-U, Nikon, Tokyo, Japan). Data were acquired and processed using Aquacosmos (Hamamatsu Photonics, Hamamatsu, Japan). Fluorescence images were obtained via alternate excitation at 340 and 380 nm, and emission signals were collected at 510 nm from all cells in the field of view using a charge-coupled device camera (ORCA-R2, Hamamatsu Photonics, Hamamatsu, Japan) coupled to an image intensifier (GaAsP, C8600, Hamamatsu Photonics, Hamamatsu, Japan). Digitized signals were stored and analyzed using the built-in programs. L-Glu (100 μM in BSS) was applied via 2 min-superfusion at a speed of 1.5 ml/min. D-(-)-2-amino-5-phosphonopentanoic acid (AP5) (100 μM in BSS) or a mixture of AP5 and 6,7-dinitroquinoxaline-2,3-dione (DNQX) (100 μM each in BSS) was applied 1 min before and during L-Glu application. Ca^2+^ increases are represented as 5-min area under the curve (AUC) for ratios of the fluorescence intensities of 340 and 380 nm from the starting point of the response.

### Cell-Surface Biotinylation Assay

Cell-surface proteins were biotinylated using the Pierce Cell Surface Protein Isolation kit (89881, Thermo Scientific, Waltham, MA, USA) according to the manufacturer’s instructions. Twelve 3.5-cm culture dishes of cells were used for each group. The cells were washed with PBS and incubated with EZ-LINK Sulfo-NHS-SS-biotin at 4°C for 45 min, followed by the addition of a quenching solution. The cells were then lysed with lysis buffer containing 1% (vol/vol) protease inhibitor cocktail set 1 (539131, Calbiochem, Darmstadt, Land Hessen, Germany). A portion of the cell lysate was saved for Western blotting (e.g., total protein fraction). The remaining cell lysate was applied to the included NeutrAvidin agarose gel. The flow-through was saved for Western blotting (e.g., intracellular protein fraction). The total proteins and intracellular proteins were mixed with 6× sodium dodecyl sulfate (SDS) sample buffer [6% (wt/vol) SDS, 30% (vol/vol) sucrose, 0.75% (wt/vol) Bromphenol Blue (BPB), 300 mM dithiothreitol (DTT) in 375 mM Tris-HCl, pH 6.8]. The biotinylated proteins were eluted with 1× SDS sample buffer [1% (wt/vol) SDS, 10% (vol/vol) glycerol, 0.125% (wt/vol) BPB, 50 mM DTT in 62.5 mM Tris-HCl, pH 6.8] (e.g., membrane protein fraction). Each fraction was then loaded onto a 7.5% polyacrylamide gel, electrophoresed, and transferred to polyvinyl difluoride (PVDF) membranes. The membranes were incubated overnight with 4% (wt/vol) BlockAce (DS Pharma Biomedical, Osaka, Japan) blocking solution at 4°C. The membranes were incubated with primary mouse monoclonal antibodies (mouse monoclonal anti-GluN1 [1:1000] [556308, BD Pharmingen, Franklin Lakes, NJ, USA], rabbit polyclonal anti-β-actin [1:5000] [ab8227, Abcam, Cambridge, UK], rabbit polyclonal anti-GluA1 [1:1000] [ab31232, Abcam, Cambridge, UK], or mouse monoclonal anti-Na^+^K^+^ ATPase [1:5000] [ab7671, Abcam, Cambridge, UK]) in Can Get Signal^TM^ solution 1 for 60 min at room temperature, followed by incubation with horseradish peroxidase (HRP)-conjugated secondary antibodies (anti-rabbit antibody [1:20,000] [GE healthcare, Little Chalfont, Buckinghamshire, UK] or anti-mouse antibody [1:20,000] [GE healthcare, Little Chalfont, Buckinghamshire, UK]) in Can Get Signal^TM^ solution 2 for 60 min at room temperature. The signals were detected with an LAS3000 (Fuji Photo Film, Co., Ltd, Tokyo, Japan) using SuperSignal West Femto Maximum Sensitivity Substrate (34095, Thermo Scientific, Waltham, MA, USA). For the relative quantification of the effects of GPC6 on the cellular distributions of GluN1 and GluA1, the signal densities in the total protein fractions, the membrane protein fractions, and the intracellular protein fractions were normalized to those of the marker proteins for respective fractions.

### MTT Reduction Assay

Cell viability was determined by 3-(4,5-dimethylthiazol-2-yl)-2,5-diphenyltetrazolium bromide (MTT) reduction activity. MTT reduction activity was measured as described previously (Abe and Matsuki, 2000; Takaki et al., 2012). Briefly, hiPSC-neurons cultured in the presence or absence of GPC6 (10 nM) were exposed to L-Glu (1, 10 mM) for 24 h. MTT was added to each well at 0.5 mg/mL and incubated for 4 h at 37°C. The culture medium in each well was carefully removed, and 50 μl of DMSO was added to dissolve the MTT formazan. The relative amount of reaction product (MTT formazan) was determined by comparing the absorbance at 570 nm (test wavelength) and 655 nm (reference wavelength) on an iMark^TM^ microplate reader (Bio-Rad, Hercules, CA, USA).

### Primary Culture of Rat Hippocampal Neurons

All animals were treated in accordance with the guidelines for the Care and Use of Laboratory Animals of the Animal Research Committee of NIHS and followed the Guide for the Care and Use of Laboratory Animals. All experimental protocols were approved by the Animal Research Committee of NIHS and conformed to the relevant regulatory standards. The brains of 1-day-old Sprague-Dawley (SD) rats were aseptically removed, and the hippocampi were dissected. The tissues were dissociated by trituration and trypsinization. After centrifugation at 1,500 rpm for 5 min, the cells were suspended in Neurobasal medium supplemented with 2% B27 and 0.1% antibiotic-antimitotic agent (NB-B27). The residual tissue aggregates were removed by filtration through a cell strainer with a pore size of 40–45 mm. The cells were plated in 8-well glass chambers (155409JP, Nunc, Waltham, MA, USA) or 96-well plates pre-coated with poly-L-lysine in the mixed medium of NB-B27 and astrocyte-conditioned medium (ACM) (1:1). The medium was changed to NB-B27 supplemented with 5 μM of cytosine arabinoside (AraC) at 1 day post-plating. One-third of the medium was changed to fresh NB-B27 each week and cells were cultured at 37°C in a humidified atmosphere containing 5% CO_2_ until 21 DIV.

### Data Analysis and Statistics

All data are presented as the mean ± SEM. Statistical analyses were performed using Tukey’s test following two-way ANOVA or Student’s *t*-test. Differences were considered significant at *p* < 0.05. The reproducibility of the data was confirmed by performing three independent experiments.

### Materials

The iCell Neurons, iCell Neuron maintenance medium, and iCell Neuron medium supplement were purchased from Cellular Dynamics International (CDI, Madison, WI, USA). Recombinant human GPC6 was purchased from R&D systems (2845-GP-050, Minneapolis, MN, USA). Fura-2 AM was purchased from Dojindo Molecular Technology Inc. (Kumamoto, Japan). The Pierce Cell Surface Protein Isolation kit (89881), SuperSignal West Femto Maximum Sensitivity Substrate (34095), maximum sensitivity substrate, BCA protein assay reagents, NB, and B27 supplements were purchased from Thermo Scientific (Waltham, MA, USA). HRP-conjugated anti-mouse and anti-rabbit antibodies were purchased from GE Healthcare Life Sciences (Little Chalfont, Buckinghamshire, UK). Anti-mouse, rabbit, goat, and chicken IgGs conjugated with Alexa Fluors were purchased from Invitrogen (Waltham, MA, USA). Poly-D-lysine, laminin, BSA, AraC, SDS, and MTT were purchased from Sigma (St. Louis, MO, USA). PFA and DNQX were purchased from Wako (Osaka, Japan). Mouse monoclonal anti-β3 tubulin was purchased from Covance (Princeton, NJ, USA). Rabbit polyclonal anti-Nestin was purchased from Millipore (Land Hessen, Germany). Chicken polyclonal anti-GFAP, rabbit polyclonal anti-β-actin, mouse monoclonal anti-pan-cadherin, rabbit polyclonal anti-GluA1, chicken anti-MAP2 antibody and mouse monoclonal anti-Na^+^K^+^ ATPase were purchased from Abcam (Cambridge, UK). Goat serum was purchased from Rockland (Pottstown, PA, USA). Hoechst 33342 was purchased from Dojindo (Kumamoto, Japan). AP5 was purchased from TOCRIS Bioscience (Bristol, UK). Protease inhibitor cocktail set 1 (539131) was purchased from Calbiochem (Darmstadt, Land Hessen, Germany). BlockAce was purchased from DS Pharma Biomedical (Osaka, Japan). Can Get Signal^TM^ was purchased from Toyobo (Osaka, Japan). Mouse monoclonal anti-GluN1 was purchased from BD Pharmingen (Franklin Lakes, NJ, USA).

## Results

**FIGURE 1:**
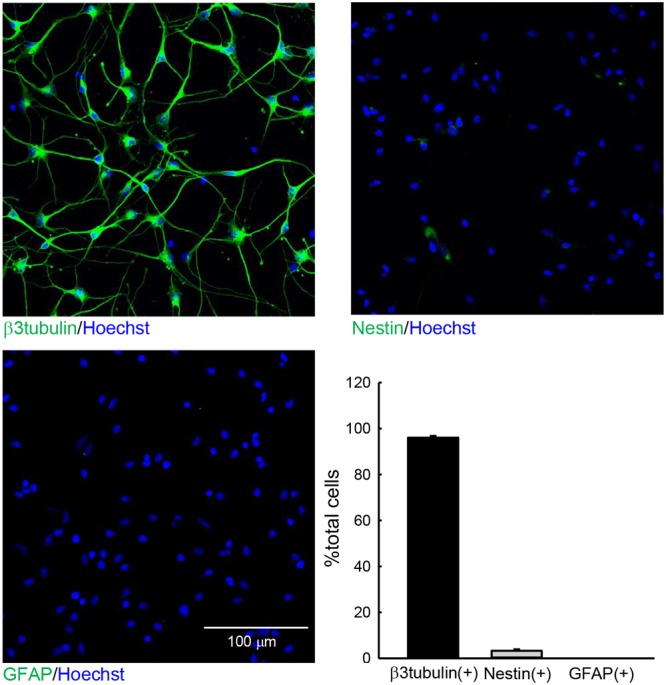
**The cells used in this study. hiPSC-neurons were immunostained with anti-β3 tubulin (top left), anti-Nestin (top right), or anti-GFAP (bottom left) antibodies and subsequently subjected to Hoechst staining at 3 DIV.** The numbers of total cells and cells that were positive for the respective differentiation markers in a 300 μm × 300 μm area were counted. The ratios of cells that were positive for respective markers are presented as the % total cells (bottom right). *N* = 6. The hiPSC-neuron sample used in study contained mostly β3 tubulin+ neurons.

We first performed immunohistochemical staining to investigate the constituents of the cells used in this study (**Figure 1**). We examined the expression of the neuronal marker β3 tubulin, the stem-cell marker Nestin, and the radial glia/astrocyte marker GFAP at 3 DIV. Among all cells, 96.05 ± 0.76% were β3 tubulin+ neurons, and 3.30 ± 0.69% cells were Nestin+. Few GFAP+ cells were observed. These data indicate that the hiPSC-neuron sample used in this study consisted primarily of neurons.

**FIGURE 2:**
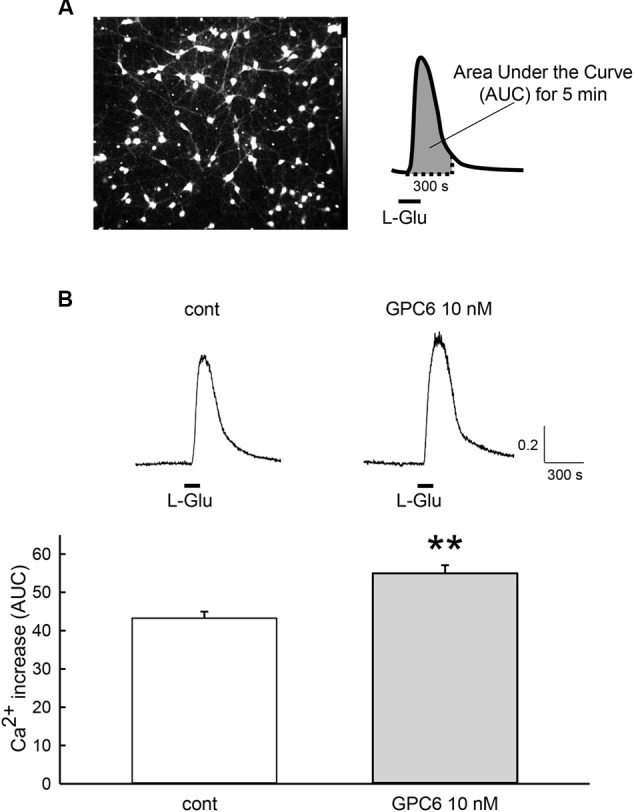
**GPC6 increased the L-Glu-induced Ca^2+^ increase in hiPSC-neurons. (A)** Left panel shows the typical control cells loaded with fura-2 AM. The 5-min area under the curve (AUC) of the ratio of the fluorescence intensities of 340 and 380 nm from the start of the response to L-Glu (100 μM, 2 min) (right panel) was quantified. **(B)** Typical Ca^2+^ traces from control and GPC6-treated cells are presented above. In the GPC6-treated group (*n* = 68), the average L-Glu-induced Ca^2+^ increase of all cells in the field of view was significantly higher than that of the control group (*n* = 53). ^∗∗^*p* < 0.01 vs. control group, Student’s *t*-test.

**FIGURE 3:**
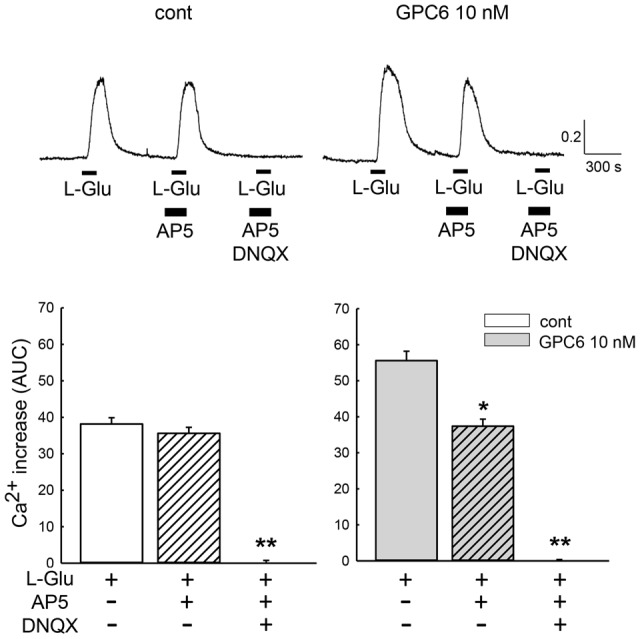
**GPC6 enhanced the NMDAR-mediated Ca^2+^ increase in hiPSC-neurons.** Typical traces of the Ca^2+^ increases in control and GPC6-treated cells are shown above. In the control group, the average value of the L-Glu-induced Ca^2+^ increase was not significantly affected by AP5 (*n* = 53). AP5+DNQX almost completely suppressed the L-Glu-induced Ca^2+^ increase. In the GPC6-treated group, AP5 significantly suppressed the L-Glu-induced Ca^2+^ increase, and AP5+DNQX almost completely suppressed the L-Glu-induced Ca^2+^ increase (*n* = 68). ^∗^*p* < 0.05, ^∗∗^*p* < 0.01 vs. AP5(-) group. Tukey’s test following ANOVA.

GPC6 has been reported to induce functional synaptogenesis in RGCs at 10 nM (Allen et al., 2012). We therefore applied 10 nM of GPC6 to the hiPSC-neurons at 3 DIV, at which point most neurites are attached to each other. After a 5-day incubation (8 DIV), L-Glu (100 μM, 2 min)-induced Ca^2+^ increases in the hiPSC-neurons were examined using fura-2 Ca^2+^ imaging. In this study, L-Glu-induced Ca^2+^ increases were quantified as the area under the curve (AUC) over a 5-min period from the start of the response (**Figure 2A**). As shown in **Figure 2B**, the average L-Glu-induced Ca^2+^ increase in all cells in the field of view was significantly enhanced in the GPC6-treated group (127 ± 4.89% of control). Representative Ca^2+^ traces from control cells and GPC6-treated cells are shown in **Figure 2B** (top panels). We next examined the contributions of NMDARs and non-NMDARs to the L-Glu-induced Ca^2+^ increase in hiPSC-neurons. In this experiment, we first examined the effect of AP5 (100 μM) and then examined the effect of a cocktail of AP5 (100 μM) and DNQX (100 μM) on the L-Glu-induced Ca^2+^ increase (**Figure 3**). In the control group, although 36.5 ± 3.69% of cells responded to AP5 (>10% suppression), the average value of the L-Glu-induced Ca^2+^ increase in all cells in the field of view was not affected by AP5. When DNQX was co-applied with AP5, the L-Glu-induced Ca^2+^ increase was completely inhibited, indicating that the L-Glu-induced Ca^2+^ increase in the control group was primarily mediated by non-NMDARs. In the GPC6-treated group, 88.5 ± 7.13% of cells responded to AP5, and the average value of L-Glu-induced Ca^2+^ increase in all cells in the field of view was significantly suppressed by AP5. When DNQX was co-applied with AP5, the L-Glu-induced Ca^2+^ increase was also completely suppressed. The top panels depict typical Ca^2+^ traces from control and GPC6-treated cells. These results indicate that GPC6 enhanced the NMDAR-mediated Ca^2+^ increase in hiPSC-neurons. The effects of GPC6 were highly reproducible, with the averaged ratios of the AP5-responsive cells (>10% suppression) in the control and GPC6-treated groups across the three experimental sessions being 26.4 ± 3.05 and 82.8 ± 10.7%, respectively (three experiments, *p* = 0.00718).

**FIGURE 4:**
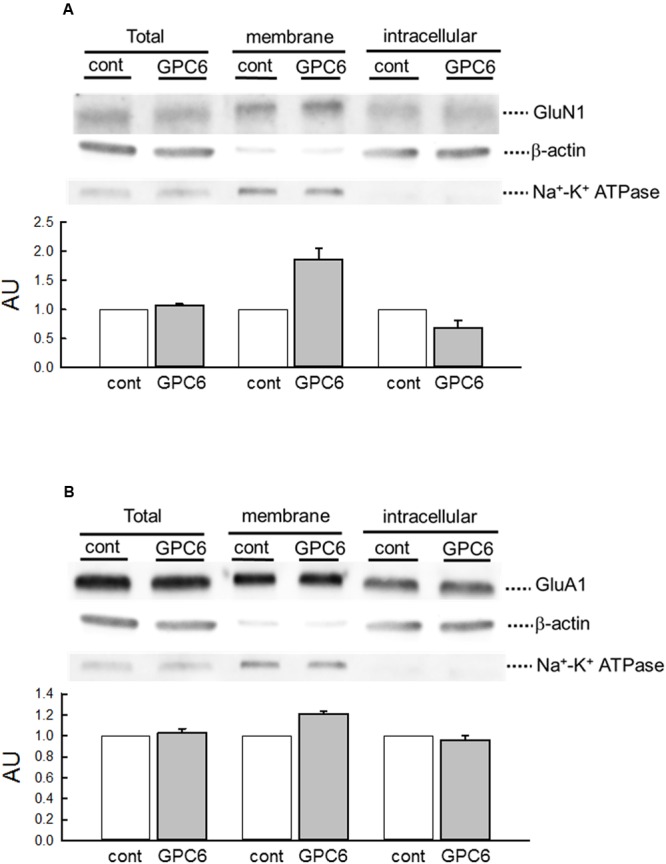
**GPC6 increased the surface levels of NMDARs of hiPSC-neurons. (A)** The expression of markers for intracellular proteins (β-actin) and membrane proteins (Na^+^-K^+^ ATPase) in respective fractions, and the cellular distribution of GluN1 (NMDARs). GPC6 induced an increase in the surface expression level of GluN1 and a corresponding decrease in intracellular expression, without little effects on the total level. **(B)** The cellular distribution of GluA1 (AMPARs). GPC6 also resulted in a slight increase in the surface expression level of GluA1, but the effect was weaker than that on GluN1. Total and intracellular GluA1 levels showed little change. The data were mean ± SE of three independent experiments.

GPC6 has been reported to increase the surface expression and clustering of the GluA1 subunit of the α-amino 3-hydroxy 5-methyl-4-isoxazolepropionic acid receptor (AMPAR) (Allen et al., 2012). We therefore examined the effects of GPC6 on the cellular distribution of NMDARs in hiPSC-neurons (**Figure 4**). In this experiment, we performed a cell-surface biotinylation experiment and quantified the expression of GluN1, which is an essential subunit for NMDAR channel activity (Meguro et al., 1992; Wenthold et al., 2003). As shown by the expression of β-actin and Na^+^-K^+^ ATPase, which marked the intracellular and membrane fractions, respectively, this protocol successfully separated membrane and intracellular proteins. GPC6 treatment resulted in increased surface expression of GluN1 (186.1 ± 19.6% of control, three experiments) and a decrease in the intracellular compartment (68.8 ± 12.7% of control, three experiments), with little effects on total GluN1 levels (**Figure 4A**). These data indicate that GPC6 increased the level of NMDARs on the cell surface of the hiPSC-neurons. We also examined the cellular distribution of GluA1 (**Figure 4B**). Although GPC6 resulted in a slight increase in the surface level of GluA1 (120.8 ± 2.850%, three experiments), the effect was weaker than that on GluN1. In addition, the intracellular and total levels of GluA1 were largely unaltered.

**FIGURE 5:**
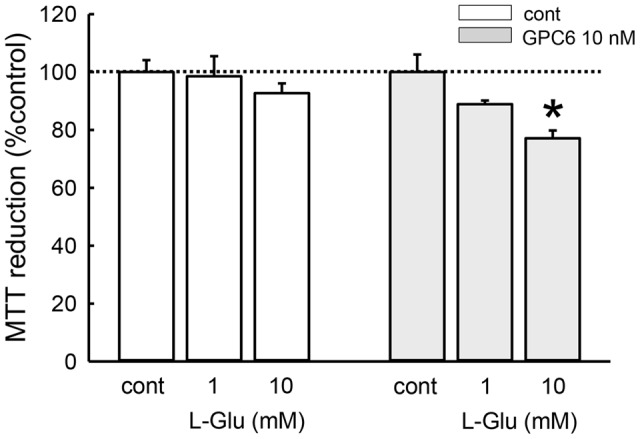
**Effects of GPC6 on the sensitivity of hiPSC-neurons to L-Glu-induced cytotoxicity.** After a 5-days treatment with GPC6, cells were exposed to L-Glu (1, 10 mM, 24 h), and an MTT reduction assay was performed. L-Glu did not cause cell damage in the control group. In the GPC6-treated group (10 nM, 5 days), L-Glu caused a concentration-dependent decrease in MTT reduction. ^∗∗^*p* < 0.01 vs. the control in each group, Tukey’s test following two-way ANOVA (*N* = 6).

We next investigated whether the sensitivity of hiPSC-neurons to L-Glu-induced cytotoxicity was affected by GPC6 treatment (**Figure 5**). Cell viability was measured using the MTT reduction assay. We first confirmed that cultured rat hippocampal neurons undergo significant damage following 24-h treatment with L-Glu at concentrations above 10 μM (Supplementary Figure S1A). After a 5-day treatment with GPC, hiPSC-neurons at 8 DIV were similarly exposed to L-Glu for 24 h. Although L-Glu did not cause cell damage in the control group, 10 mM L-Glu caused a significant decrease in MTT reduction in the GPC6-treated group. Although the sensitivity to L-Glu was lower in hiPSC-neurons than in cultured rat hippocampal neurons, GPC6 treatment nevertheless rendered hiPSC-neurons sensitive to L-Glu-induced cytotoxicity. The GPC6-induced increase in surface NMDAR expression may be involved in the increased sensitivity to L-Glu.

## Discussion

Since the successful neuronal differentiation of hiPSCs was first reported (Takahashi et al., 2007; Dimos et al., 2008), hiPSC-neurons have been expected to improve the prediction accuracy of the effects of new candidate compounds in humans during preclinical drug development, and the extent of functional maturation determines the application of hiPSC-neurons. In recent reports, Dage et al. (2014) revealed that hiPSC-neurons express various ion channels and receptors through a multitude of methods (patch-clamp, calcium flux, extracellular recordings, etc.). However, the number of cells that responded to NMDA was still low at 7 DIV (Dage et al., 2014). In the present study, we clarified that the astrocyte-derived GPI-anchored HSPG GPC6 increased the cell-surface NMDAR expression in hiPSC-neurons, rendering them sensitive to excitotoxicity.

### The Significance of Functional NMDA Receptor Expression in hiPSC-Neurons

*N*-methyl-D-aspartate receptors have been implicated as mediators of excitotoxicity associated with many neurological disorders, including ischemia, epilepsy, brain trauma, dementia, and neurodegenerative disorders (Doble, 1999; Kemp and McKernan, 2002). Larger quantities of Ca^2+^ ions move through NMDARs, and excessive Ca^2+^ increases due to NMDARs lead to excitotoxicity (Ferreira et al., 1996; Stout et al., 1998). More recently, increased attention has been placed on indirect increases in NMDAR function to alleviate psychiatric disorders such as schizophrenia (Field et al., 2011). Thus, NMDAR ligands, both inhibitors and potentiators, are expected to be beneficial in the treatment of several neurological and psychiatric conditions. However, the therapeutic ratio for most NMDA antagonists is poor, with significant adverse effects at clinically effective doses (Gardoni and Di Luca, 2006). Our present findings will help expand the study of the pharmacology of human NMDARs at the preclinical stage. In this study, L-Glu-induced damage reached significance at 10 mM in GPC6-treated hiPSC-neurons. This effective concentration is higher than that for cultured rat hippocampal neurons. The L-Glu-induced Ca^2+^ increase in the GPC6-treated hiPSC-neurons is much lower than that in the cultured rat neurons (**Figure 3**; Supplementary Figure S1B), suggesting that the expression levels of L-Glu receptors in hiPSC-neurons are still too low to cause L-Glu cytotoxicity comparable to rat hippocampal neurons. The specific composition of NMDAR subunits might also be involved in the insufficient Ca^2+^ increase. NMDARs are heterotetramers of two GluN1 subunits and two GluN2 subunits (Cull-Candy et al., 2001). Because GluN2B-containing NMDARs exhibit slower kinetics than GluN2A-containing receptors (Williams et al., 1993), GluN2B-containing NMDARs play a larger role in excitotoxicity than GluN2A-containing NMDARs (Zhou and Baudry, 2006).

### Potential of Glial Factors to Enhance the Functional Maturation of hiPSC-Neurons

Recent studies have shown that interactions with glial cells are indispensable for the functional maturation of newborn neurons (Allen, 2013; Corty and Freeman, 2013; Shigemoto-Mogami et al., 2014). Huge numbers of astrocytes are generated just prior to the postnatal wave of synaptogenesis and play important roles in synaptogenesis in the developing brain (Wang and Bordey, 2008). As such, some reports have demonstrated that co-culturing with rodent astrocytes promotes the functional maturation of hiPSC-neurons (Shcheglovitov et al., 2013; Tang et al., 2013; Odawara et al., 2014). However, the efficiency of the astrocyte feeder depends on culture viability. Identifying the factors responsible for the effects of the astrocyte feeder will allow the functional differentiation of hiPSC-neurons to be controlled more easily. GPC6 was originally demonstrated to increase the surface expression and clustering of AMPARs in RGCs, thereby promoting synaptogenesis (Allen et al., 2012). However, the precise pathway by which GPC6 exerts these effects remains to be elucidated. Allen et al. (2012) also have suggested that GPCs act via the interaction of HS with AMPARs rather than through the delivery of the associated morphogens. The HSPG Syndecan-4 is known to be necessary for the trafficking of transglutaminase-2 to the cell surface (Scarpellini et al., 2009). HSPGs might commonly regulate the membrane trafficking of functional proteins. GPC6 may also suppress NMDA receptor internalization, thereby leading to increased cell-surface expression. Unfortunately, spontaneous Ca^2+^ responses that reflect functional synaptogenesis were not obtained in this study. The ratio of astrocytes was almost 0% in our hiPSC-neuron cultures, which is uncommon in primary cultures of rodent neurons. The astrocyte cell surface may contain additional key molecules that further enhance functional maturation.

### The Functional Maturation Time of hiPSC-Neurons

Increasing evidence suggests that the functional maturation of hiPSC-neurons takes longer than that of rodent neurons. Shi et al. (2012) reported that a 45–100-days differentiation period is necessary to successfully record miniature excitatory post-synaptic currents (mEPSCs) from hiPSC-neurons. Moreover, hiPSC-derived neurons transplanted into mice brains took several months to establish functional synapses with the host circuitry, suggesting that the rate of functional synaptogenesis is an intrinsic property of hiPSC-neurons (Shi et al., 2012; Espuny-Camacho et al., 2013). A recent report suggested that the human-specific duplication of the SLIT-ROBO Rho GTPase activating protein 2 (SRGAP2) gene extends the phase of spine development and thereby causes ‘spine neoteny,’ which allows human cortical neurons to receive a significantly higher number of synaptic inputs (Charrier et al., 2012). Taken together, these data suggest that the long time period required for the functional synaptogenesis of hiPSC-neurons may be related to species differences. To improve the usability of hiPSC neurons for drug development, functional synaptogenesis over a shorter period is needed; however, the protocols for accelerating neuronal maturation should be chosen carefully. Our present data highlight the promise of glial factors, and glial factors that are actually active during brain development may be the first choice to accelerate the functional maturation of hiPSC-neurons for drug-development applications.

## Author Contributions

KS designed this work and wrote the paper. KS, KT, YS-M, and KC performed the experiments and analyzed the data. KS, KT, YS-M, KC, and YS joined discussions and revised the paper. All authors have approved the present version of the manuscript and have agreed to be accountable for all aspects of the work regarding questions related to the accuracy or integrity of any part of the work.

## Conflict of Interest Statement

The authors declare that the research was conducted in the absence of any commercial or financial relationships that could be construed as a potential conflict of interest.
