# Supplementary material for: Glypican 6 Enhances N-Methyl-D-Aspartate Receptor Function in Human-Induced Pluripotent Stem Cell-Derived Neurons
Source: Front Cell Neurosci. 2016 Nov 15;10:259. doi: 10.3389/fncel.2016.00259 (PMC5108764; doi:10.3389/fncel.2016.00259)
Supplement: Supplementary file 1 [file Presentation_1.pdf]

Supplementary Material

Glypican 6 enhances N-methyl-D-aspartate receptor function in human-induced pluripotent stem cell-derived neurons

Kaoru Sato*, Kanako Takahashi, Yukari Shigemoto-Mogami, Kaori Chujo, Yuko Sekino

*** Correspondence:** Kaoru Sato: kasato@nihs.go.jp

1 Supplementary Data

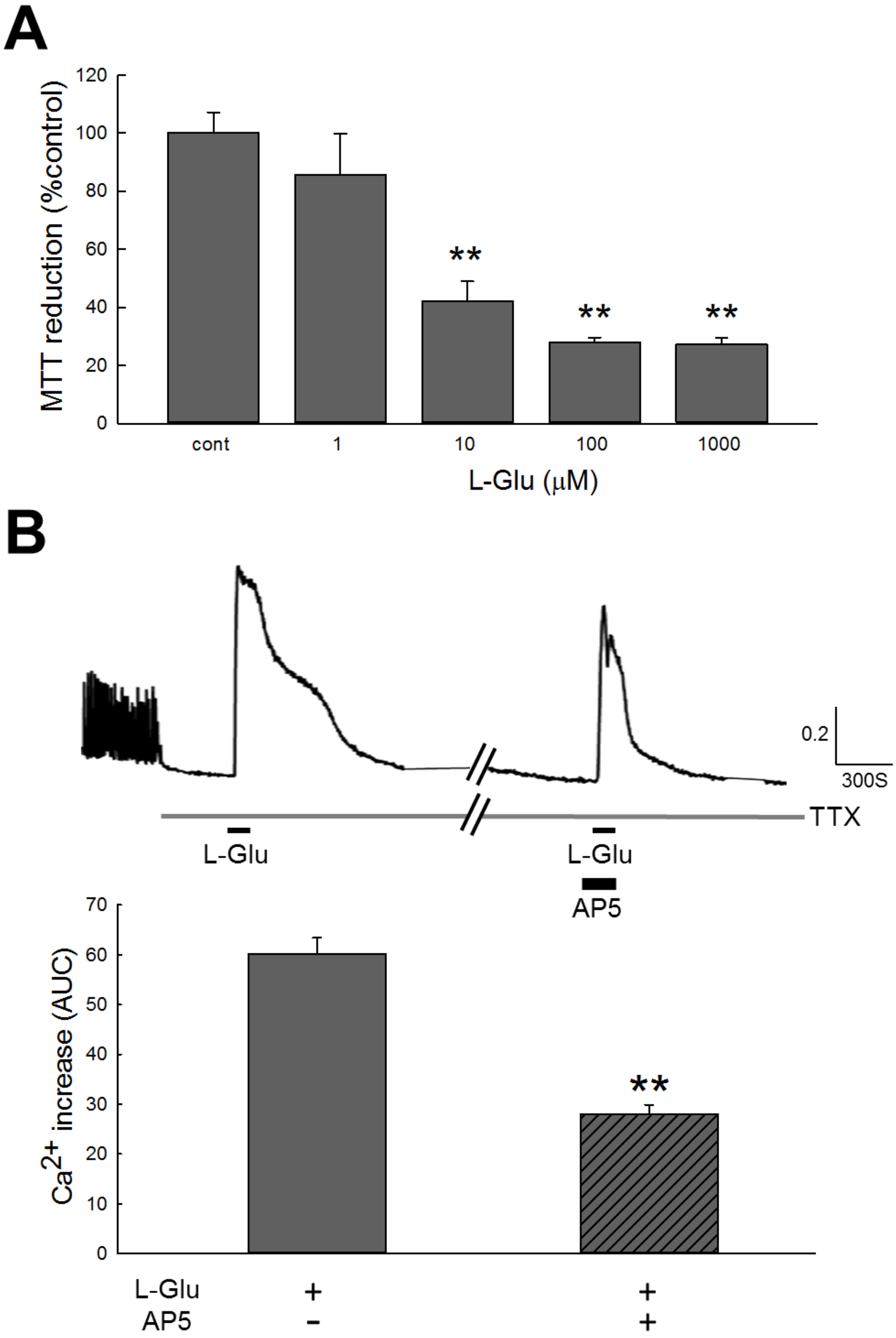

Supplementary Figure 1. L-Glu-induced cytotoxicity and the contributon of NMDARs to the L-Glu-induced Ca^{2+} increase in cultured rat hippocampal neurons

A. Cultured rat hippocampal neurons at 21DIV were exposed to L-Glu (1 μM-1 mM, 24 hr), and an MTT reduction assay was performed. L-Glu caused significant cell damage at concentrations above 10 μM. **: p<0.01 vs. control, Tukey's test following ANOVA (N=6).

B. Typical traces of the L-Glu-induced Ca^{2+} increase in the absence and presence of AP5 are shown above. Spontaneous activities were observed before the application of TTX. The effects of AP5 (100 μM) and AP5+DNQX (100 μM) on the L-Glu-induced Ca^{2+} increases in cultured rat hippocampal neurons (n=78) at 21DIV were examined by fura-2 imaging. AP5 significantly suppressed the L-Glu-induced Ca^{2+} increase. **: p<0.01 vs. AP5(-) group. Student's *t* test.
